# A randomized controlled study for Yuanhu Zhitong dropping pills in the treatment of knee osteoarthritis

**DOI:** 10.1097/MD.0000000000020666

**Published:** 2020-06-12

**Authors:** Yubiao Gu, Jin Huang, Honggang Guo, Xuewen Song, Jianguo Li, Yanlong Shi, Xingwen Xie

**Affiliations:** a Gansu Provincial Hospital of TCM; b Gansu University of Chinese medicine; c Department of Orthopaedics, Affiliated Hospital of Northwest Minzu University; d Department of Orthopaedics, Gansu Second Provincial People's Hospital, Lanzhou, Gansu, China.

**Keywords:** protocol, Yuanhu Zhitong dropping pills, Knee osteoarthritis

## Abstract

**Background:**

Knee osteoarthritis (KOA) is a common chronic disorder of knee and the leading cause of pain in the elderly with an overall prevalence of 50% in people over 60 years of age. This disease is an important factor affecting the quality of life of middle-aged and elderly people, and its main symptom is knee joint pain. Due to the pain, the knee joint activity function is limited, bringing great pain to patients, affecting their quality of life, effective prevention, and treatment of KOA is a modern medical problem.

**Methods:**

The 60 patients who met the inclusion criteria were randomly divided into the treatment group and the control group. In this study, single center, randomized control and equivalent clinical trial were used for treatment. The treatment group received Yuanhu Zhitong dropping pills within 4 weeks, and the control group received diclofenac sodium sustained-release capsule treatment within 4 weeks. The main measures were visual analogue scale (VAS), WOMAC osteoarthritis index score and gastrointestinal symptoms rating scale (GSRS).Secondary measures included biochemical markers and adverse reactions during treatment.

**Result:**

The results of this trial will be published on the website of China Clinical Trial Registration Center (http://www.chictr.org.cn/searchprojen.aspx) and in peer-reviewed journals or academic conferences.

**Conclusions:**

This study is to assess the efficacy and safety of Yuanhu Zhitong dropping pills for knee osteoarthritis (KOA).

**Registration:**

PROSPERO (registration number ChiCTR1900024712).

## Abstract 2

Supplemental Digital Content is available in the text

## 1 Introduction

Knee osteoarthritis (KOA) is a common chronic knee disease, with pain and limited function as the main causes.^[1,2]^ More than 9,000,000 people have been diagnosed with KOA through clinical and imaging methods in USA.^[3]^ In China, 19.4% of the elderly population more than 60 years of age suffer from KOA.^[4,5]^ According to the Bulletin of the World Health Organization, osteoarthritis is predicted to be the fourth leading cause of disability by 2020.^[6]^ Patients often have symptoms such as pain and dysactivity, which limits the mobility of patients, cause psychosocial problems such as low self-efficacy and depression, and decreases the quality of life.^[7,8]^ The current therapeutic for KOA were mainly aimed to reducing pain and improvement of joint mobility.^[9,10]^

According to the recent guidelines, knee replacement surgery was the only effective pain management approach for the end-stage KOA.^[11,12]^ For the non-surgical treatment of KOA, pharmacological, non-pharmacological and alternative treatments were widely applied in the clinical practice.^[13]^ Among them pharmacologic treatments were the mostly widely adopted. For example acetaminophen, topical treatment, nonsteroidal, anti-inflammatory drugs (NSAIDs), COX-2 inhibitors, opioids and intra-articular cartilage-protective agents.^[14]^ However, long time taking anti-inflammatory drugs was associated with serious adverse reactions, such as digestive and renal impairment.^[15]^ A number of patients had to stop taking drugs for the side effects. Most patients are unwilling to receive routine treatment due to side effects, contraindications, and unsatisfactory relief of symptoms.^[16]^ while not all the patients are the ideal surgical candidates, some patients afraid of operation. Therefore non-invasive, safe and effective treatment options are in great demand for those patients to improve symptoms.

Chinese medicine has been used to manage osteoarthropathy for thousands of years. Yuanhu Zhitong dropping pills were also applied for treat KOA. However its efficacy and security has not been reported in the literature. The purpose of this study is to assess the efficacy and security of Yuanhu Zhitong dropping pills for KOA, a large sample size and a reliable blind method will be used to draw a reliable conclusion.

## 2 Methods

### 2.1 Study design

This study will be a single-blinded, randomized, and Diclofenac Sodium Sustained Release Capsules controlled trial. It will be performed at Gansu Provincial Hospital of Traditional Chinese Medicine, Lanzhou, China. The project was evaluated and approved by the Research Ethics Committee of Gansu Provincial Hospital of Traditional Chinese Medicine (Ethical batch No. FJ/04-IRB/C/018-V3.0, Ethics Review Adoption Form places in the Supplemental Digital Content). We planned to begin recruiting patients from July 2019, Patients with stage I, II, and III knee osteoarthritis will be recruited. In this study, there will be 8 investigators. Including 1 chief orthopedic physician, 5 orthopedic physicians, 2 outcome assessors. Five orthopedic physicians will recruit patients. The included patients should meet the inclusion criteria and will introduce the patient to the trial process. The 5 orthopedic physicians will also tell the participants possible benefits and risks.

### 2.2 Participants

The following eligibility criteria apply to all patients for recruitment into the study. Patients will be recruited through the Orthopaedics clinic of Gansu Provincial Hospital of Traditional Chinese Medicine. After oral explanation and reading of the procedures used in the study, patients agreeing to participate will sign an informed consent form.

Inclusion criteria:

1. Aged 45 to 70 years;
2. Meet the diagnostic criteria for knee osteoarthritis according to American Rheumatism^[17]^ Association guidelines;
3. Visual analogue scoring (VAS) is more than 10 mm and less than 60 mm;
4. Patients with stage I, II, and III knee osteoarthritis;
5. No medication has been taken in the past 2 weeks.

Exclusion criteria:

1. Undergone knee surgery;
2. traumatic osteoarthritis;
3. Pregnant and lactating women;
4. Unable to attend the follow-up within 1 month;
5. Complicated with liver, kidney, hematopoietic system, digestive system, cardiovascular and cerebrovascular diseases, and other serious primary diseases;
6. Tuberculosis of knee joint, deformity of knee joint, and malignant tumors of knee joint;
7. Psychiatric diseases and can not cooperation;
8. Opioid analgesics, sedatives and hypnotics, and alcohol abuse;
9. Allergy to experimental drugs.

### 2.3 Study interventions

#### 2.3.1 Treatment group

Treatment group Patients will oral Yuanhu Zhitong dropping pills, 10 pills, 3 times a day. The treatment period will last 4 weeks. Drugs are provided by Gansu Longshen Rongfa Pharmaceutical Company. The approval is No. Z20010024.

#### 2.3.2 Control group

Control group Patients will oral diclofenac sodium sustained-release capsules 100 mg once per day, drugs are provided by Xiansheng Pharmaceutical Company. The treatment period will also 4 weeks. The approval is H20023856.

### 2.4 Sample size calculation

Preliminary estimates put the number of cases in each group at 25. Considering the influence of shedding and elimination (20%), the overall sample size was set as 60 cases, 30 cases in the experimental group and 30 cases in the control group.

### 2.5 Randomization and allocation concealment

Sixty patients will be divided into Yuanhu Zhitong dropping pills and Diclofenac Sodium Sustained Release Capsules groups with a ratio of 1:1, random number table will be used for divide the groups. Only the chief orthopedic physicians will be allowed to know the differences of the groups.

### 2.6 Single-blinding

Treatment and Control practitioners were not provided details of treatment plans or grouping information within the envelope in advance. No participant or relevant medical personnel was authorized to interfere with individual treatment selections. Evaluators are blinded to the treatment each patient has received, their function being to assist each patient in completion of the various questionnaires and scoring the respective scales. It is intended that the independent biostatisticians are also blinded when conducting the statistical analysis.

### 2.7 Discontinuation criteria

1. Occurrence of anaphylaxis or severe adverse events;
2. It was found in the study that the therapeutic effect was too poor to have clinical value;
3. It is difficult to evaluate the curative effect because of the important deviation in the design or implementation of the clinical research scheme.

### 2.8 Outcomes

#### 2.8.1 Primary outcomes pain^[18,19]^

When the patient takes the medicine, it will be recorded as the first day. Patients will mark on a 100 mm visual analogue scale to assess the pain of their knee (from no pain to severe). At the end of 1, 2, 3, and 4 week, patients themselves and the assessors will record it.

#### 2.8.2 Secondary outcomes

##### 2.8.2.1 Knee function and patient satisfaction

Knee function will be measured The Western Ontario and McMaster Universities Osteoarthritis Index (WOMAC),^[20,21]^ patients themselves and the assessors will also record it at the end of 1, 2, 3, and 4 week. We will record the patient satisfaction at the time their treatment period is finished. A questionnaire will be applied. The questionnaire will be divided into 4 stages, including very satisfied, satisfied, just like, and not satisfied. Patients will fill in the questionnaire by themselves.

### 2.9 Complications and adverse events during and after treatment

All expected and unexpected adverse events will be recorded during practitioner contact time throughout the study. The adverse events associated with both drugs are usually the possible occurrence of the immune system during treatment (rash, macular rash, urticaria, allergic reaction); Central system (dizziness); Digestive system (nausea, vomiting, diarrhea or abdominal discomfort); Respiratory system (difficulty breathing); Cardiovascular system (chest tightness, palpitations, shortness of breath); Others (dry mouth, fever, chills). All serious adverse events will be reported immediately to the study contact for further investigation of the cause.

### 2.10 Data management

Data will be collected by 2 independent trained research assistants using Microsoft Excel. Final data of research data after completion will be collected. A formal data monitoring committee will not be required for the reason the risk is minimal. Independent investigators will monitor the data periodically.

### 2.11 Statistical analysis

All data collected during the study will input into SPSS 21.0 statistical software for statistical analysis. Among them, the counting data are expressed in the form of number of individuals and percentage (%). The measurements are expressed in the form of mean and standard deviation (X ± S), and processed by non-parametric test or paired *t* test of 2 related samples. In the statistical results, if *P* < .01, there was a significant difference in the cue data, if *P* > .01, there was no significant difference in the cue data.

### 2.12 Ethics and dissemination

This trial was approved by Ethics Committee of Gansu Traditional Chinese Medicine Hospital.

Written inform consent will be obtained from all participants or their authorized agents. We will provide free drugs for patients in case and control group and strictly confidential the research data. The result of the trial will be presented on the website of the Chinese Clinical Trial Registry and published in open access journals.

## 3 Results

The results of this trial will be published on the website of the China Clinical Trial Registration Center and in peer-reviewed journals or academic conferences.

## 4 Discussion

The results showed that the Yuanhu Zhitong Drop Pill group was superior to the Western medicine group in terms of VAS scores, WOMAC scale assessment results, patient satisfaction, and gastrointestinal symptoms assessment scale (GSRS scale),^[22]^*P* < .05, adverse reactions. The number of cases was significantly less than that of the western medicine group. This shows that Yuanhu Zhitong drop pills can effectively relieve limb pain without side effects, can improve the quality of life of patients, and has a significant effect.

In recent years, with the continuous development of traditional Chinese medicine research, it has achieved significant results in the treatment of knee osteoarthritis. At the same time, with its unique advantages such as multiple targets for therapeutic effects and low toxic and side effects, it has obtained a large number of patients and doctors. There are still many deficiencies in the research of traditional Chinese medicine for knee osteoarthritis. There are some limitations with this study. First, the sample size of this study is small and it is a single-center randomized controlled study. Second, patients using VAS scores are too subjective. Third, a large number of clinical studies lack long-term efficacy. The safety of the drug need long time to observe.

Therefore, large-scale randomized double-blind controlled experiments should be carried out in future clinical research to strive for innovative and prospective related studies with rich center points and large sample sizes. In conclusion, we hope that the results of this study can provide new evidence for the treatment of early knee osteoarthritis pain with traditional Chinese medicine.

## Author contributions

**Conceptualization:** Yubiao Gu, Jin Huang.

**Data curation:** Yubiao Gu, Jin Huang.

**Formal analysis:** Xuewen Song.

**Investigation:** Xuewen Song, Jianguo Li.

**Methodology:** Yubiao Gu, Jin Huang, Honggang Guo.

**Project administration:** Honggang Guo, Xingwen Xie.

**Resources:** Xuewen Song, Yanlong Shi.

**Software:** Jianguo Li.

**Supervision:** Honggang Guo, Xingwen Xie.

**Validation:** Honggang Guo, Xingwen Xie.

**Writing – original draft:** Honggang Guo, Yubiao Gu, Jin Huang.

**Writing – review and editing:** Honggang Guo, Xingwen Xie.

## Supplementary Material

Supplemental Digital Content
