# Supplementary figures and images for: A randomized controlled study for Yuanhu Zhitong dropping pills in the treatment of knee osteoarthritis
Source: Medicine (Baltimore). 2020 Jun 12;99(24):e20666. doi: 10.1097/MD.0000000000020666 (PMC7302597; doi:10.1097/MD.0000000000020666)

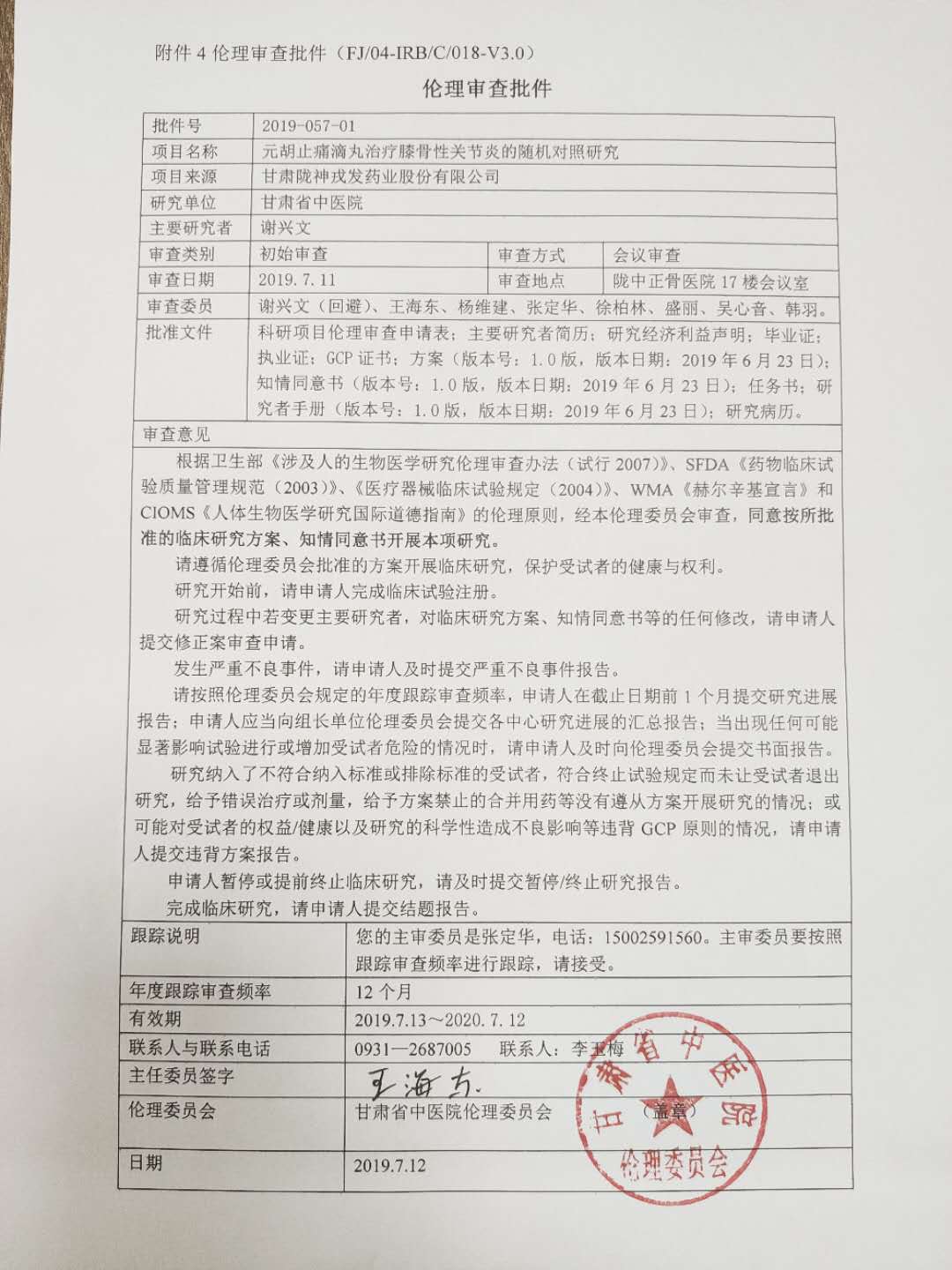

Supplement: Supplemental Digital Content [file medi-99-e20666-s001.jpg]
